# Pharmacologic Profile of A_2A_ Adenosine Receptors: Identifying Patients with Intermittent Claudication and Associated Myocardial Ischemia

**DOI:** 10.1016/j.cjco.2025.06.024

**Published:** 2025-07-23

**Authors:** Pierre Deharo, Julien Fromonot, Soumeya Aliouane, Marion Marlinge, Bouchra Talbi, Nathalie Kipson, Tristan Werquin, Julia Dodivers, Thomas Cuisset, Marine Gaudry, Régis Guieu, Franck Paganelli

**Affiliations:** a Cardiology Department, Timone University Hospital, Marseille, France; b Centre for Cardiovascular and Nutrition Research, INSERM (French National Institute of Health and Medical Research), INRAE (French National Research Institute for Agriculture, Food, and Environment), Aix Marseille University, Marseille, France; c Laboratory of Biochemistry, Marseille University Hospital (AP-HM), Marseille, France; d Department of Vascular Surgery, Timone University Hospital, Marseille, France; e Department of Cardiology, North Hospital, Marseille, France

**Keywords:** intermittent claudication, myocardial ischemia, adenosine A2A receptors, biological markers

## Abstract

Low-extremity peripheral artery disease (LE-PAD) is often associated with coronary artery disease (CAD). Development of biomarkers is needed to identify those among LE-PAD patients who have associated CAD. The pharmacologic profile of adenosine A_2A_ receptors (A_2A_R; expression, cyclic adenosine monophosphate [cAMP] production, half maximal effective concentration [EC_50_]) evaluated on peripheral blood mononuclear cells is useful because these parameters are modified during myocardial ischemia. A total of 127 patients were included; 75 with CAD had a positive flow-fraction-reserve (FFR) but no intermittent claudication. Among those with LE-PAD, 27 had a positive FFR, and 25 had a negative FFR. The A_2A_R expression and EC_50_ were lower in patients with a positive FFR vs a negative FFR. Obstructive CAD might be detected by measuring the adenosine A_2A_R profile.

## Résumé

La maladie artérielle périphérique des membres inférieurs (MAPMI) est souvent associée à la coronaropathie. Il est nécessaire d’établir des biomarqueurs pour distinguer, parmi les patients atteints de MAPMI, ceux qui présentent également une coronaropathie. Il est utile de connaître le profil pharmacologique des récepteurs de l’adénosine A_2A_ (expression et production d’AMP cyclique, concentration efficace médiane [CE_50_]) évalué sur cellules mononucléées du sang périphérique (CMSP), car ces paramètres sont modifiés en cas d’ischémie myocardique. Un total de 127 patients a été inclus, dont 75 atteints de coronaropathie ayant obtenu un résultat positif à l’évaluation de la réserve de flux fractionnaire (FFR pour *Flow-Fraction-Reserve*) mais ne présentant aucune claudication intermittente. Parmi les patients atteints de MAPMI, 27 ont obtenu un résultat positif à l’évaluation de la FFR et 25, un résultat négatif. L’expression des récepteurs de l’adénosine A_2A_ et la CE_50_ étaient plus faibles chez les patients ayant obtenu un résultat positif à l’évaluation de la FFR que chez ceux ayant obtenu un résultat négatif. Il est possible de détecter une coronaropathie obstructive en mesurant le profil des récepteurs de l’adénosine A_2A_.

Low-extremity peripheral artery disease (LE-PAD) often is associated with coronary artery disease (CAD), and these patients share the same risk factors. However, due to adverse effects, exploration of LE-PAD patients by invasive coronary angiography (ICA) and percutaneous coronary intervention is not recommended. The presence of peripheral artery disease is associated with higher rates of post–percutaneous coronary intervention death and myocardial infarction.^1^ In addition, coexistence of CAD and peripheral artery disease significantly impact patient outcomes.^2^ Hence, development of biomarkers is needed to identify among LE-PAD patients those who have associated significant CAD. In this context, the pharmacologic profile of adenosine A_2A_ receptors (A_2A_R; which are implicated in the control of coronary blood flow^3^) evaluated on peripheral blood mononuclear cells (PBMCs) is useful because their behavior mirrors that of A_2A_Rs from coronary and peripheral arteries, and A_2A_R properties are modified during chronic myocardial ischemia.^4^

## Hypothesis

We hypothesized that CAD and/or LE-PAD may influence the A_2A_R pharmacologic profile. Thus, we aim to evaluate A_2A_R properties (A_2A_R expression and cyclic adenosine monophosphate [cAMP] production in response to agonist stimulation) in patients with intermittent claudication. This could help in identifying those who, in addition to peripheral arteriosclerosis, have significant CAD.

## Patients and Methods

Patients with either LE-PAD with planned ICA, or ICA for stable angina without LE-PAD were recruited in North Hospital and Timone Hospital in Marseille, France (Department of Cardiology and Vascular Surgery) during the period from January 2023 to July 2023. A total of 127 patients were included (mean age 68.2 ± 10 years); 93 were men (73%), and 34 were women (26.7%). ICA followed by flow fraction reserve (FFR) measurement was performed in all patients. A total of 75 CAD patients had an FFR < 0.8 but without intermittent claudication. All LE-PAD patients were scheduled for surgery. Among LE-PAD patients undergoing vascular surgery, 27 had an FFR < 0.8, and 25 had an FFR > 0.8.

Data were expressed as means and standard deviations or as medians and ranges. The characteristics were compared using the Mann-Whitney *U* test. Receiver operating characteristic curves were established to define the best threshold value for A_2A_R expression and half maximal effective concentration (EC_50_) to discriminate between patients with vs without CAD. A_2A_R expression on PBMCs was evaluated using western blot (arbitrary units [AU], as previously described).^5^ cAMP production (ie, EC_50_ cAMP production) was evaluated via immunoassay, after incubation of PBMCs (7.5 X 10^5^ cells for each point), with increasing concentration of Adonis, an antibody with agonist properties.

## Results

**Figure 1:**
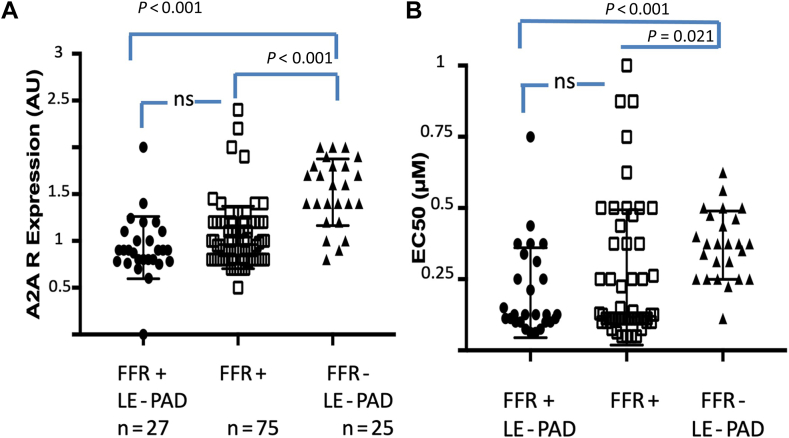
A_2A_ adenosine receptors (A_2A_R) profile of patients with low-extremity peripheral artery disease (LE-PAD), with or without associated coronary artery disease. (**A**) A_2A_R expression evaluated on peripheral blood mononuclear cells. (**B**) Evaluation of cyclic adenosine monophosphate production (see Methods). EC_50_ was defined as the half maximal production of cyclic adenosine monophosphate. AU, arbitrary unit; FFR, flow fraction reserve; ns, nonsignificant.

Median A_2A_R expression was lower in patients with an FFR < 0.8, vs in those with an FFR > 0.8 (Fig. 1A). The presence of associated LE-PAD did not significantly change A_2A_R expression.

The receiver operating characteristic curves indicate that with a cutoff equal to 1.15 AU, the sensitivity was 75%, with a specificity of 84%, whereas with a cutoff of 1.35 AU, the sensitivity was 90%, with a specificity of 76%. The area under the curve was 0.89, *P* < 0.0001.

The median EC_50_ was significantly lower in patients with an FFR < 0.8 vs those with a negative FFR (Fig. 1B). The presence of associated LE-PAD did not significantly influence the median EC_50_ value. A cutoff equal to 0.23 μM gave a sensitivity and specificity of 72%. The area under the curve was 0.83, *P* = 0.004.

The main finding of this study is that low A_2A_R expression and/or a low EC_50_ value, in patients with LE-PAD, is associated with the presence of significant CAD. The low EC_50_ is associated with the presence of A_2A_R reserve, which was attributed to an adaptive mechanism to ischemia.^4^^,^^5^

These findings support the possibility that obstructive CAD can be detected noninvasively by measuring the expression and function of PMBC A_2A_Rs, and this approach should be evaluated in a larger cohort.
